# Common Structural Patterns in the Maxicircle Divergent Region of Trypanosomatidae

**DOI:** 10.3390/pathogens9020100

**Published:** 2020-02-05

**Authors:** Evgeny S. Gerasimov, Ksenia A. Zamyatnina, Nadezda S. Matveeva, Yulia A. Rudenskaya, Natalya Kraeva, Alexander A. Kolesnikov, Vyacheslav Yurchenko

**Affiliations:** 1 Faculty of Biology, M. V. Lomonosov Moscow State University, Moscow 119991, Russiaaak330@yandex.ru (A.A.K.); 2 Martsinovsky Institute of Medical Parasitology, Tropical and Vector Borne Diseases, Sechenov University, Moscow 119435, Russia; 3 Institute for Information Transmission Problems, Russian Academy of Sciences, Moscow 127051, Russia; 4 Life Science Research Centre, Faculty of Science, University of Ostrava, 710 00 Ostrava, Czech Republic

**Keywords:** divergent region, maxicircle, kinetoplast, trypanosomatids, repeats, genomic rearrangements, mitochondrion

## Abstract

Maxicircles of all kinetoplastid flagellates are functional analogs of mitochondrial genome of other eukaryotes. They consist of two distinct parts, called the coding region and the divergent region (DR). The DR is composed of highly repetitive sequences and, as such, remains the least explored segment of a trypanosomatid genome. It is extremely difficult to sequence and assemble, that is why very few full length maxicircle sequences were available until now. Using PacBio data, we assembled 17 complete maxicircles from different species of trypanosomatids. Here we present their large-scale comparative analysis and describe common patterns of DR organization in trypanosomatids.

## 1. Introduction

Trypanosomatids are unicellular parasitic organisms with a single large mitochondrion per cell [1,2]. Mitochondrial DNA of trypanosomatids is a network of concatenated circular molecules of two types: maxicircles and minicircles [3]. Minicircles are exclusive to trypanosomatids, these relatively small, but numerous, molecules are directly involved in U-insertion/deletion editing system as they encode gRNAs [4,5]. There are thousands of minicircles present in a single mitochondrion [6,7,8]. In contrast, maxicircles are much larger (25–50 kbps) and present in up to 100 copies [9]. It is highly likely that both molecule populations are heterogeneous in the cell [10,11,12,13,14,15,16,17]. Maxicircles are functional equivalents of mitochondrial genome of other eukaryotes. Mitochondrial genes are compactly located in the so-called coding region (CR) of a maxicircle, which is typically about 16 kbps, and demonstrate high level of synteny between species [18,19,20,21]. The rest of maxicircle’s molecule is termed the divergent region (DR), sometimes also called the variable region [22,23,24,25].

The DR was shown to be composed of repetitive sequence elements and, therefore, extremely hard to sequence and assemble [26,27,28]. Most of the previous comparative studies were based not on the direct nucleotide sequence analysis, but rather on hybridization techniques, which allowed to estimate sequence homology between fragments of maxicircles only roughly [19,25,29]. These experiments showed that the DR sequences of maxicircles vary significantly between species/strains, while, in contrast, the CRs preserve a high level of synteny. The nature of this variation remained unclear. Only few fragments from DR were directly sequenced following PCR amplification, molecular cloning and Sanger sequencing. For example, the analysis of 2.76 kb fragment from DR of *Leishmania tarentolae* revealed that various repetitive elements are grouped into larger clusters, which, in turn, are repeats themselves. Most repetitive units within clusters are not perfect repeats, as they share 65–100% sequence identity [26]. The DR was proposed to regulate gene expression, however, a putative origin of replication, topoisomerase II binding sites, and heterogeneously sized transcripts were documented in this region, arguing that its role may be more complex [22]. 

With the development of sequencing technologies some maxicircles were sequenced completely [12,30,31,32]. However, even paired-end Illumina sequencing was often not sufficient to assemble the complete maxicircle, and, because of that, most studies were focused only on coding region with short flanks [11,13,18], and even the most recent assembly from Illumina data had recovered only 3.5 kb of DR (as in [32]). Analysis of 3.5-kb and 5-kb regions of DR of *Trypanosoma lewisi*, flanking 12S and ND5 genes, respectively, revealed that these loci have different patterns of repeats [31]. Keeping up with authors’ terminology, we will call them sections I and II, respectively. Section I is composed of short highly repetitive units, while section II is a series of tandem duplications of a longer sequence. The repeat units in DR tend to be organized in a head-to-tail orientation, frequently forming nearly perfect clusters of tandem repeats.

Arguably, an even more interesting observation is the ability of DR to evolve quickly. Massive structural rearrangements were observed in cultures of *Leishmania* spp. under various conditions [33,34,35]. Of note, molecular studies of these rearrangements have never been done with full length maxicircle sequences.

Repeated sequences, especially tandem repeats and large duplications, complicate the assembly process and result in errors even in the coding sequences. Nevertheless, sufficiently long reads can help to overcome this hurdle [36]. In the current work, we used PacBio reads to assemble maxicircles, thus, contributing to the collection of known DR structures. We present here sequences of 17 new full-length maxicircles, more than double of what has been available before, and brief comparative analysis of their DR structures, revealing common patterns and specific features of this region.

## 2. Results

### 2.1. Complete Maxicircle Assembly Overview

Using our custom pipeline to assemble PacBio reads containing 12S and/or ND5 mitochondrial genes we recovered 17 full-length mitochondrial maxicircle sequences of different trypanosomatid species. 

Maxicircles have different sizes. The shortest sequence in this study of 23,201 bp belongs to *Trypanosoma brucei* (Lister 427), while the longest one of 47,384 bp is from *T. cruzi* (Dm28c). Table 1 demonstrates that size variation of maxicircles primarily reflects the differences in length of the DR, which is consistent with previous observations [18,19,21]. The CRs of all assembled maxicircles are similar in length and exhibit a high level of synteny. Some minor length variations between the CRs can be explained by the editing patterns (variable length of the edited domains) of some cryptogenes. As such, the shortest CR sequences were documented in *Trypanosoma* spp., where cryptogenes undergo the most extensive RNA editing [4,5], while the longest CR sequences were in monoxenous trypanosomatids, whose cryptogenes have reduced editing domains [37,38,39].

The DRs of maxicircles are much more variable in size, reflecting the dynamics of this structure in evolution. This difference is over 20 kbps for maxicircles of different trypanosomatid species. Even within the phylogenetically compact group of *Leishmania* spp., the DR length between the two strains of *L. braziliensis* and three strains of *L. donovani* vary by ≈3 kbps. 

### 2.2. Nucleotide-Level Analysis of the DR Sequences

**Figure 1:**
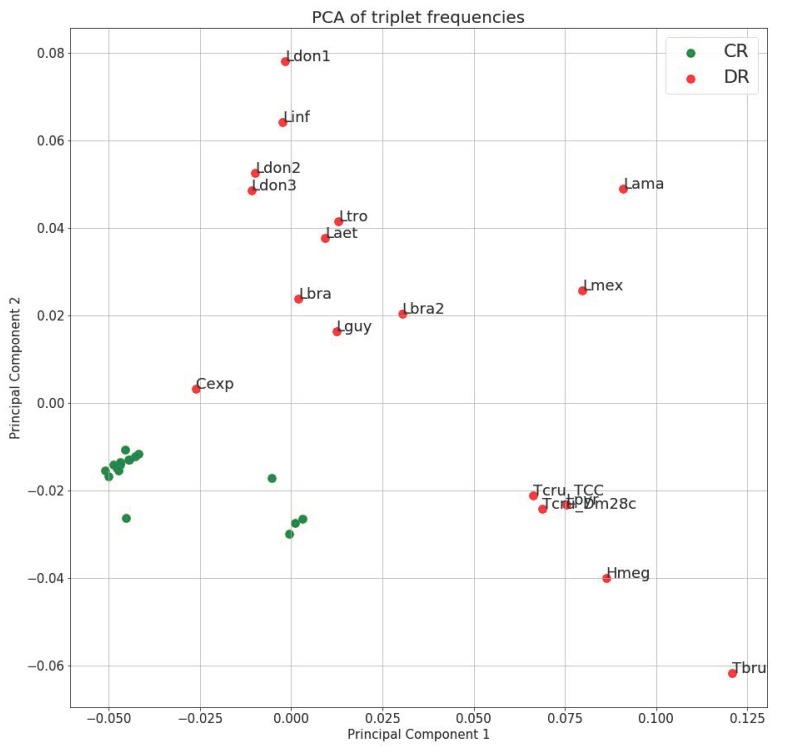
Principal component analysis of triplet spectra of maxicircles. Triplet frequency vectors were calculated for the coding region (green) and divergent region (red) of each maxicircle. One and two principal components were chosen to show on the scatter plot. Lama = *Leishmania amazonensis*; Hmeg = *Herpetomonas megaseliae*; Laet = *Leishmania aethiopica*; Cexp = *Crithidia expoeki*; Lbra2 = *Leishmania braziliensis* (208-905); Lbra = *Leishmania braziliensis* (208-954); Ldon1 = *Leishmania donovani* (Pasteur); Ldon2 = *Leishmania donovani* (193-S616); Ldon3 = *Leishmania donovani* (FDAARGOS_361); Lguy = *Leishmania guyanensis*; Linf = *Leishmania infantum*; Lmex = *Leishmania mexicana*; Lpyr = *Leptomonas pyrrhocoris*; Ltro = *Leishmania tropica*; Tbru = *Trypanosoma brucei*; Dm28c = *Trypanosoma cruzi* (Dm28c); TCC = *Trypanosoma cruzi* (TCC).

PCA (Principal Component Analysis) of triplet frequencies demonstrated that even on the level of very small sequence elements (triplets) the DR has species-specific composition and the clustering is generally consistent with phylogenetic relationships of the species (Figure 1). Importantly, despite the documented length variation between strains and species, their triplet spectrum remains very stable. For example, while the length variation between the DRs of *Leishmania donovani* strains and *L. infantum* is about 4500 bp, they cluster together on the PCA plot. The same is true for *T. cruzi* strains, which possess even more dramatic DR length difference. The most parsimonious explanation for this observation is that the level of repeat arrays is very unstable, despite the fact that the structure of small repetitive units of the DR is species-specific. Rearrangements at this level may account for the DR length variation and can happen frequently, due to the recombination between stable repetitive units.

**Figure 2:**
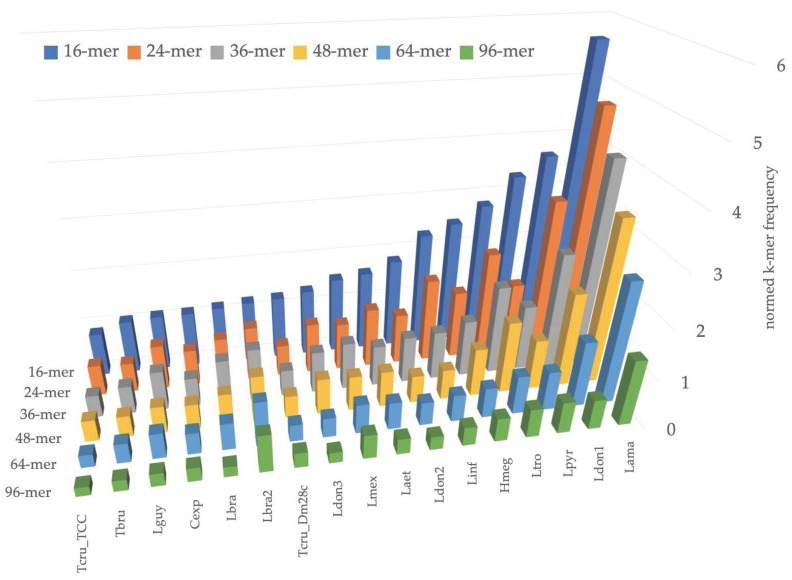
Number of occurrences of most frequent k-mer in maxicircle for values of k = 16, 24, 36, 48, 64, and 96. Species abbreviations are the same as for Figure 1.

Next, we analyzed the pattern of k-mer frequencies in maxicircles for small values of k (Figure 2). This analysis revealed the regularity of DR structure at a very basic level of short perfect repeats. For example, *Leishmania amazonensis* has 160 exact matches of 5’-TTAAATTAAATTAAATTAAATTAAA-3’ sequence in the 33,779 bp-long maxicircle, and this sequence is a pentamer of the ‘TTAAA’ block itself. The DR of *L. pyrrhocoris* H10 has only 45 perfect repeats of 5’-ATATTGAAAATAAAGTGCTAGATA-3’ sequence in its maxicircle of approximately the same length, and this sequence is not composed of smaller units. In the DRs of *T. cruzi* TCC maxicircles, the most frequent 24-mer is repeated only 25 times, implying more diffused organization of repeated units.

Such a small-scale regularity appears to be changing quickly and does not reflect phylogenetic relationships between species. Three *L. donovani* strains have maxicircles, which are located close to each other on the PCA axis (Figure 1), exhibit different patterns of k-mer occurrences (Figure 2).

### 2.3. Large-Scale Divergent Region Architecture 

Previous studies of sequences, derived from the DRs of *Leishmania* spp., revealed that they are composed almost exclusively of repeats. These repeats are grouped in arrays or clusters [26,40]. In addition to *Leishmania*, these were also studies in *Strigomonas oncopelti* (called then *Crithidia oncopelti*) [41]. Here, we built full maps of divergent region of 17 different species using the MUMmer package to identify homologous regions and to show global patterns of DR organization.

**Figure 3:**
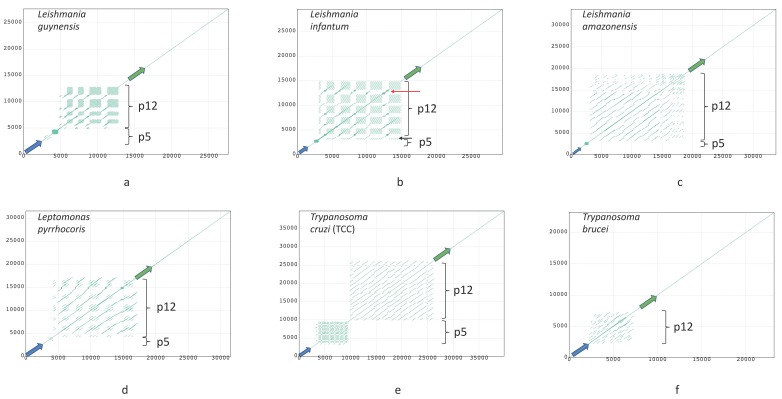
Dotplots of maxicircles of *Leishmania guyanensis* (**a**); *L. infantum* (**b**); *L. amazonensis* (**c**); *Leptomonas pyrrhocoris* (**d**); *Trypanosoma cruzi* strain TCC (**e**); *T. brucei* (**f**). Green and blue arrows denote *12S rRNA* and *ND5* genes, respectively. All plots show full maxicircle starting with the ND5 gene at (0,0). Red and black arrows in (**b**) indicate the I-element and a single-copy array repeat unit, respectively. Dot plots of other assembled maxicircles can be found in Figure A1.

**Figure A1:**
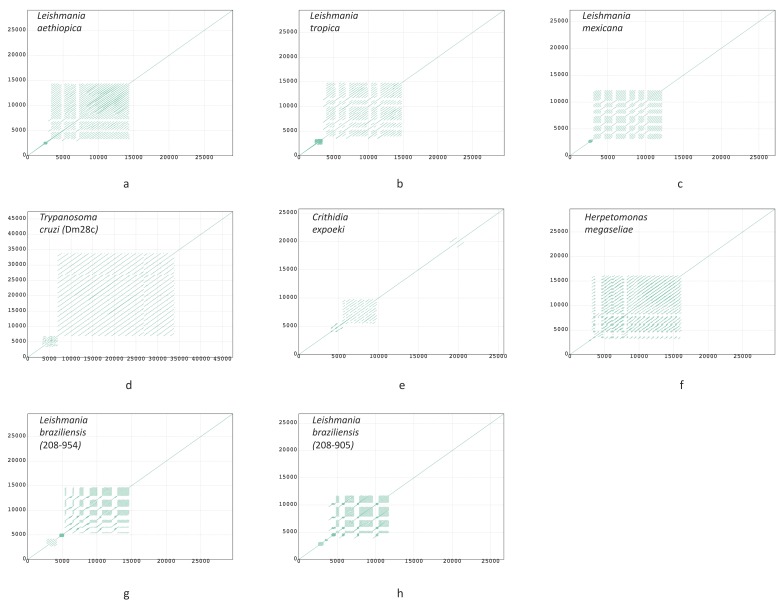
Dotplots of maxicircles of Leishmania aethiopica (**a**); L. tropica (**b**); L. mexicana (**c**); Trypanosoma cruzi strain Dm28c (**d**); Crithidia expoeki (**e**); Herpetomonas megaseliae (**f**); L. braziliensis strain 208-954 (**g**); L. braziliensis strain 208-905 (**h**). All plots show full maxicircle starting with the ND5 gene at (0, 0).

Dotplots of six maxicircles, depicting variants of DR organization in studied species, are shown in Figure 3. Dotplots of other assembled maxicircles are shown in Figure A1. There are some common traits of the DR architecture. Firstly, repetitive elements are always arranged in a head-to-tail way. Secondly, there are two elements, which can be usually distinguished in the DR sequences. Hereafter, we will denote them as P5 and P12, according to their proximity (P) to the *ND5* and *12S* genes, respectively. Both elements are composed of repeated sequences, but these sequences have a different length of the repeat (periodicity), structure, and pattern of repetitions. The P5 is a tandem repeat with a large period, but small number of repetitions. In contrast, the P12 element is composed of highly repetitive units with a small period, which are organized in repeat arrays of varying length. Arrays are frequently interspersed by repeat units of a radically different type, the I-elements, which are dissimilar to repeats forming the arrays (Figure 3b, red arrow). The I-elements are common in *Leishmania* maxicircles, where they clearly demarcate arrays’ borders. According to the number of arrays in the P12 element, the DRs can be pentameric (*Leishmania infantum* or *Leptomonas pyrrhocoris*; see Figure 3b,d), or tetrameric (*Leishmania guyanensis*, see Figure 3a). In some species the I-element is absent. In these cases, arrays of repeats are not visually separated on dotplots and appear as single-tandem clusters (for example, the P12 element of *L. amazonensis* and *T.cruzi*, Figure 3c,e, respectively). Such clusters, however, are not perfect tandem repeats, as the repeated units diverge with varying percent of sequence identity.

**Figure A2:**
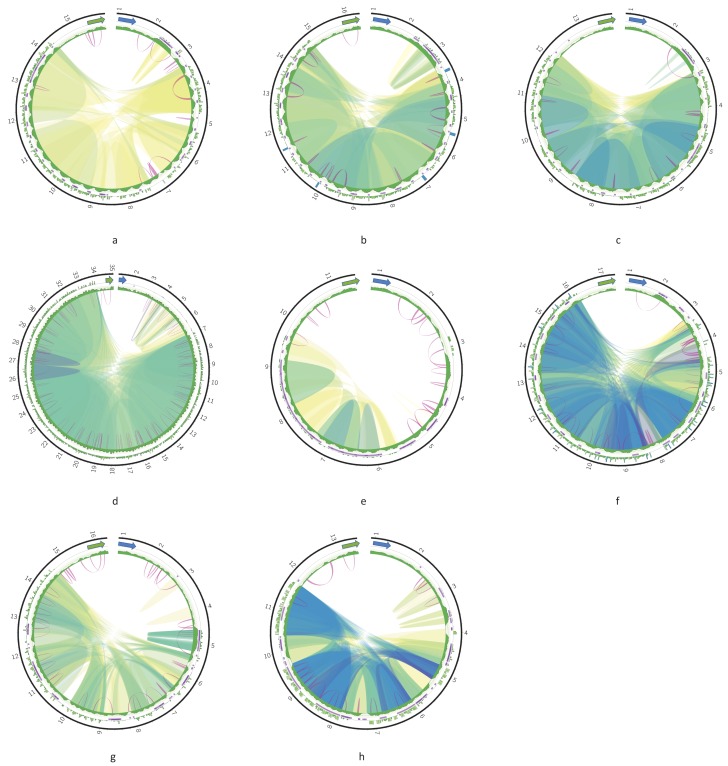
Circos plots of the DR of Leishmania aethiopica (**a**); Leishmania tropica (**b**); Leishmania mexicana (**c**); Trypanosoma cruzi strain Dm28c (**d**); Crithidia expoeki (**e**); Herpetomonas megaseliae (**f**); Leishmania braziliensis strain 208-954 (**g**); Leishmania braziliensis strain 208-905 (**h**). Green and blue arrows indicate 12S and ND5 genes, respectively. The outer track is a histogram of 24-mer frequency; the middle track shows tandem repeats; the inner track is a GC content profile. Ribbons inside the circle connect homologous regions; the color represents percent of sequence identity in the range [80%; 100%]. Violet arcs denote inverted repeats.

The general architecture of the DR, described above, however, undergoes major changes in some species. In *T. brucei*, which seems to have rather small DR, the P5 element is reduced to a short non-repetitive sequence. Very similar reduction of the P5 element was also documented in *Herpetomonas megaseliae* (Figure A2c). 

**Figure 4:**
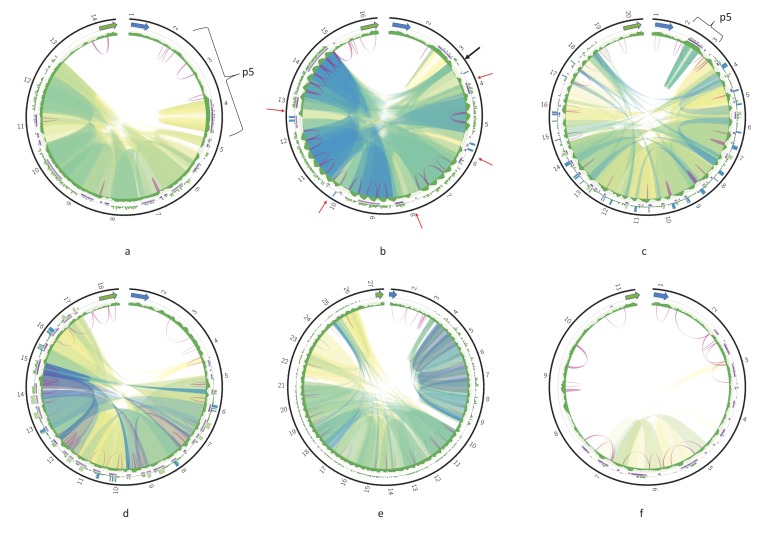
Circos plots of the divergent region (DR) of *Leishmania guyanensis* (**a**); *L. infantum* (**b**); *L. amazonensis* (**c**); *Leptomonas pyrrhocoris* (**d**); *Trypanosoma cruzi* strain TCC (**e**); *T. brucei* (**f**). Green and blue arrows denote the *12S rRNA* and *ND5* genes, respectively. Outer tracks are histograms of 24-mer frequency; middle tracks indicate tandem repeats; and the inner tracks are GC-content profiles. Ribbons inside the circle connect homologous regions; color represents percent of sequence identity in range [80%; 100%] in the order red, yellow, green, and blue. Violet arcs denote inverted repeats.

The complete maps of the DRs (Circos plots) for the six species are shown in Figure 4; maps for the rest of the assembled genomes are presented in Figure A2. The repeats have various degree of sequence similarity. This can be exemplified by the P12 element of, for example, *Leishmania guyanensis* (Figure 4a) or *L. aethiopica* (Figure A2a). The most striking case in this regard is *T. brucei* (Figure 4f), in which the sequence similarity between most repeats is below 70%, with only few repeat units in the middle of the DR sharing about 90% of sequence identity. The P5 element of most *Leishmania* spp. ends with an almost perfect tandem repeat, which is very GC-rich and is dimeric in most genomes. A single copy of array repeat unit at the beginning of the P12 element is present in all *Leishmania* genome analyzed. This unit is marked by the black arrows in Figure 3b and Figure 4b. 

In contrast, there are some species with almost identical regions in their DRs. For example, five arrays of repeats from the pentameric P12 element of *Leishmania infantum* share over 95% identity in their sequences (Figure 4b). In *L. amazonensis*, highly similar blocks of much smaller size are also detected in the P12 element (Figure 4c).

Of note, the GC profiles of most *Leishmania* spp. DRs are very regular (e.g., the repeat arrays within the P12 element have a distinct pattern), while three *Trypanosoma* spp. and three monoxenous trypanosomatids analyzed here have less regular GC profiles of their P12 element.

Repeat units within arrays in the P12 element can form either perfect or separated by short indels tandems. Interestingly, no tandems can be detected in both *T. cruzi* maxicircles (Figure 4e and Figure A2d) even with rather relaxed mreps settings. Inverted repeats could be detected in all DR sequences. There was no discernable common pattern of their localization. Almost invariably, these repeats are very short with identity between complementary parts reaching 85–91%. Very frequently, the inverted repeats were detected only in some copies of repeat units within arrays, and were missing from neighboring units because of the sequence divergence. 

Special kinds of sequences in the DR of some species can be revealed with the k-mer frequency plots. Blue bars on outer track histograms in Figure 4 indicate that a 24-mer starting at the current sequence position is repeated over 40 times in *Leishmania infantum*, *L. amazonensis*, and *Leptomonas pyrrhocoris*. These are always extremely AT-rich sequences composed of a single repeated motif. Interestingly, unique non-repetitive sequences can also be present in the DRs, mostly on the border between the P5 and P12 elements. 

### 2.4. Comparative Analysis and Dynamics of DR Structure 

**Figure 5:**
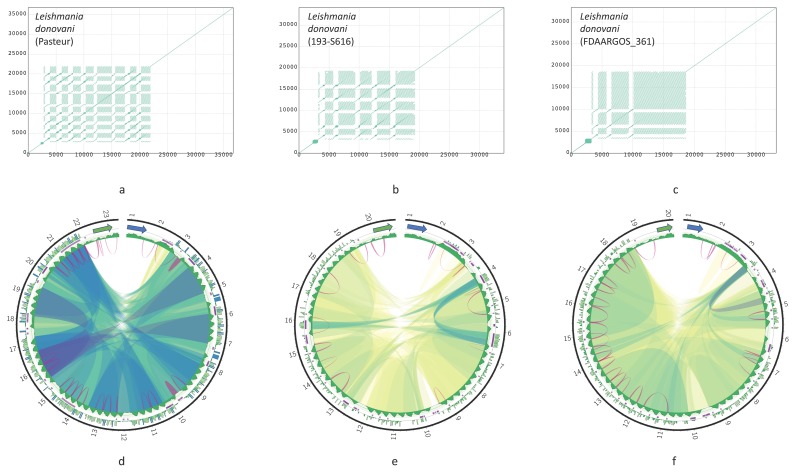
Dotplots and corresponding Circos plots of the DR of *Leishmania donovani* strains: Pasteur (**a**,**d**); 193-S616 (**b**,**e**); FDAARGOS_361 (**c**,**f**) Circos plots are presented in the same way as in Figure 4.

Structural rearrangements in the DRs have been described earlier [33,34,35]. Here, we compared the full DRs of maxicircles in order to reveal structural variations between strains and species. The DR structures for three strains of *L. donovani* are presented in Figure 5. The P12 element is octameric, pentameric, and trimeric in the Pasteur, 193-S616, and FDAARGOS_361 strains, respectively. Both the number of arrays in the P12 element and the size of each array vary significantly. Most proximal to the *12S* rRNA gene array in the FDAARGOS_361 strain is big, while in the Pasteur strain all eight arrays are rather small in size. Thus, the P12 element appears mainly responsible for the overall maxicircle size variation. 

Sequence similarity between units of arrays also differ between three *L. donovani* strains. For example, in the Pasteur strain, all eight repeat arrays are almost identical (Figure 5d), while in the 193-S616 strain most repeats share less than 90% identity, with only three blocks of tandem repeats having higher similarity (Figure 5e, blue ribbons).

**Figure 6:**
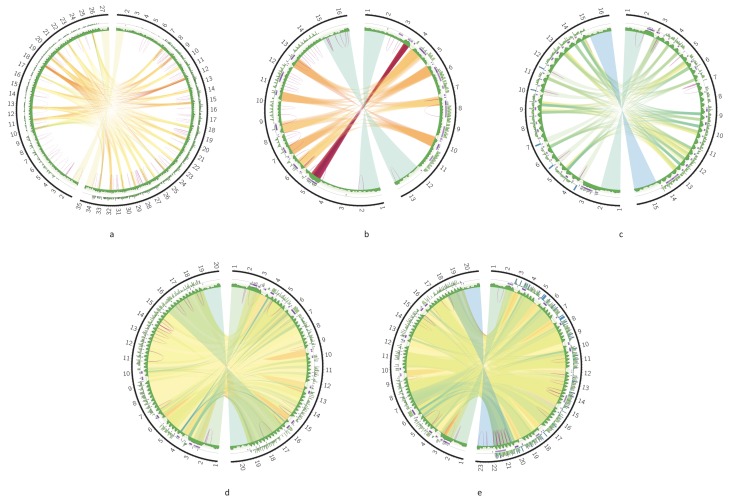
Circos plots comparing the DRs of two species or strains: *T. cruzi* strains TCC (left contig) and Dm28c (right contig) (**a**); *L. braziliensis* strains (**b**); *Leishmania tropica* (left contig) and *L. aethiopica* (right contig) (**c**); *L. donovani* strains 193-S616 (left contig) and FDAARGON_361 (right contig) (**d**); *L. donovani* strains 193-S616 (left contig) and Pasteur (right contig) (**e**).

Next, we compared the DR structures in different strains and species in order to reveal most conservative sequence elements. In all the cases, the highest similarity was between the *12S* rRNA and *ND5* genes, flanking the DR sequences. In addition, there were also sequence elements inside the DR, which shared over 80% identity between the strains (Figure 6a,b) or even the species (Figure 6c). These elements were usually localized in the P12 element of the DR, and only in *L. braziliensis* low similarity was detected between tandem repeats of the P5 element. The locations of homologous elements in the P12 coincide with the layout of repeat arrays, indicating that repeat arrays are built from repeat units with different degrees of conservation. More conserved units tend to be in the beginning of each array. Different strains of *Leishmania donovani* demonstrate the highest degree of sequence similarity in their DRs (Figure 6d,e).

### 2.5. Quality Control of Maxicircle Assemblies

Since PacBio sequencing datasets, used in this study, targeted primarily nuclear genomes and, therefore, were not enriched for mitochondrial DNA, we occasionally observed low coverage of the final maxicircle assembly (below 10 reads). Inspecting coverage profiles in Integrated Genome Browser (IGB), we noticed that generally, but not always, less covered regions of the maxicircle were from the DR. We carefully examined all the assemblies and discarded one for *L. panamensis*, which did not meet our quality criteria. Two maxicircle contigs (marked in Table 1) were not circularized by the Canu assembler, but had a detectable sequence overlap, which included a copy of the *ND5* gene and this allowed us to close the circle correctly. 

For two species (*L. donovani* FDAARGOS_361 and *L. pyrrhocoris* H10) we used available Illumina paired-end sequencing reads to assess the quality of assemblies. We found that Illumina reads align well on both of our assemblies, supporting all nucleotides of the maxicircle sequences. For *L. donovani* strain FDAARGOS_361, mapping the paired-end reads (150 + 150 with average insert size of 180 bp) onto our assembly produced a profile with uneven coverage of the maxicircle sequence. For *Leptomonas pyrrhocoris* H10 we used the 150 + 150 paired-end reads with average insert size of 940 bp. In this case the coverage profile did not differ between the coding and divergent regions [42] (Supplementary Figure S1). We explain this by the cumulative effect of the sequencing insert size (longer reads improve repeat resolution) and the complexity of the DR (the *L. donovani* DR has more homogeneous repeat units and, as such, is a more difficult reference for the read mapping). 

We also compared the maxicircle of *Leishmania donovani* strain Pasteur with that from the GenBank (CP022652), which was produced by the HGAP3 assembly algorithm from the same PacBio reads. The GenBank sequence contains duplicated nucleotides on the 5’ end, which overlap with ≈4000 bp on the 3’ end of the assembly. After trimming, both sequences had nearly equal length and high level of sequence identity, though identity percent dropped slightly in the DR, indicating that the choice of the PacBio assembly method may influence the resultant sequence, especially in the low-covered regions. Supplementary Figure S2 demonstrates synteny of both assemblies.

## 3. Discussion

Here we present the sequences of 17 new full trypanosomatid maxicircles. The big problem of maxicircle sequencing is the divergent region, which remains difficult to assemble even using the modern NGS techniques [31,32]. In the current work we used PacBio datasets to assemble maxicircles, but even with this technique the assemblies often suffered from the low coverage in their DR. Nevertheless, in most cases we had a sufficient number of reads to reconstruct the sequence of a full maxicircle. 

Our analysis revealed the common architectural pattern of the DR organization. We described two regions with different structure, the P5 and P12 elements, according to their proximity to the *ND5* and *12S* genes, respectively. Previously, similar structures have been described for *T. lewisi* [19] as sections I and II [31]. The section I (which is P12 in our terms) was composed of short, highly repetitive units; while section II (P5 in our terms), consisted of several large duplications. This appears to be a common architecture of the DR of maxicircles. 

The difference in DR length is connected to the massive rearrangements in the P12 element, which is built from the repetitive elements, further organized in arrays. Numbers and lengths of such arrays are species-specific and can change quickly. Sequence rearrangements in the *12S*-proximal clusters of the *Leishmania* spp. DRs were investigated previously [34,40] and the current work confirms previous findings, stipulating that the P12 compartment is very dynamic. Of note, differences in the P12 region also determine DR length variation in different isolates of *Trypanosoma brucei* [22]. The driving forces of such quick rearrangements remain to be investigated further. While the mechanism of these changes remains unclear, the DR will stay unreliable as a putative genetic marker for trypanosomatid identification, because its stability in isolated cultures over time has never been investigated.

It has been also proposed that there can be different structural classes of maxicircles within the same pool of kDNA [10,18,34], but our findings can neither confirm, nor disprove this. In most cases we might have insufficient coverage to detect alternative maxicircle variants that can be present, taking into account that the proportion of alternative variant can be as low as 2%. On the other hand, our results confirm that the *12S*-proximal region is very dynamic, varying between phylogenetically close strains of *Leishmania donovani* and *L. braziliensis*. Inverted repeats are detected frequently in this region, possibly indicating its high recombination potential. Moreover, we detected a few PacBio reads bearing *ND5* and/or *12S* rRNA sequences, which were not included in the final assembly by the software used. These reads can originate from rare maxicircle variants, but current datasets have insufficient sequencing depth to confirm this.

Repeat units in the P12 locus are both species-specific and conservative, strongly supporting the conclusion that these arrays are not junk DNA. It is plausible to suggest that these repeat arrays contain binding sites for some transcription factors or DNA maintenance proteins, which may stabilize such DNA-protein complexes. In this case, structural rearrangements of the P12 element can play a role in nucleoid remodeling, controlling access of proteins to the coding region loci. The P5 element appears to be more conservative in terms of organization, and in *Leishmania* spp. it is present in a form of a GC-rich tandem repeat. In some species it can be reduced or absent, indicating that this structural pattern is not crucial for maxicircle replication or transcription.

It has been suggested that a putative replication origin and promotor sequences are located in the DR [12,22]. For example, imperfect palindrome sequences and motifs with high sequence similarity to the CSB3 blocks of minicircles were documented in some repeat copies of the *T. brucei* P12 element [22]. It has been proposed that they can serve as maxicircle replication origins. In our work, we did not detect any sequence homology with minicircular CBS3 blocks in the DRs of assembled maxicircles. We did detect various inverted repeats and imperfect palindromes, scattered in the DR, but their exact biological role remains to be investigated further. 

## 4. Materials and Methods 

### 4.1. Complete Maxicircle Assembly

In order to assemble full-length maxicircles, we used the available PacBio datasets deposited in the SRA NCBI database. Information on accession numbers of sequences, used in this work, is summarized in Table 2. 

Reads were downloaded and converted to the fasta format. For quality control, all reads below 10 kbps were discarded. A local database for Blastn (from local installation of NCBI-Blast suite 2.3.0+) was built from these reads and searched for the *12S* and *ND5* maxicircle gene sequences, using sequences of *Leptomonas pyrrhocoris*, *Trypanosoma cruzi, T. brucei*, *Leishmania amazonensis*, *L. tarentolae*, and *Crithidia expoeki* as queries. Parameters for Blastn search were “-evalue 1e-10 -word_size 5 -outfmt 6”. Reads producing alignments with alignment length over 1000 were selected for further analysis. Assembly was performed with the Canu v1.8 assembler [44] with the “genomeSize = 50 k”. For *Leishmania donovani* and *Leptomonas pyrrhocoris* H10, we also tested the assembly protocol with different values of the “genomeSize” parameter (in a range of 20–100 k with a step of 10 k) and it did not influence the final contig sequence. To eliminate overlaps, the resultant sequences were trimmed using the nucmer tool from MUMmer v3.23 package [45] with settings “nucmer -maxmatch -nosimplify contigs.fasta contigs.fasta; show-coords -lrcTH out.delta”. 

### 4.2. Maxicircle Annotation

Assembled maxicircles were annotated in a semi-automatic mode. We checked the synteny of maxicircle genes using Blastn searches for *ND1*, *ND4*, *COI*, *Cyb*, *9S*, *MURF2*, and *COII* genes. These genes are not (or almost not) edited and, therefore, are good markers for automated annotation. We concluded that all assembled maxicircles preserved gene order documented for trypanosomatid maxicircles [21].

The best scoring match was used to determine the initial annotation coordinates for the *12S* and *ND5* genes, followed by the manual curation. For the convenience of further analysis, we rotated all maxicircle sequences, so that each assembly started from the *ND5* gene, followed be the DR, *12S*, and *rps12* genes. 

### 4.3. Quality Control of the Assemblies

Assemblies were checked by two criteria: (i) read coverage is sufficient throughout the assembly, and (ii) final maxicircle has perfectly assembled CR. The PacBio reads were mapped on the assembly with bwa mem algorithm of bwa v0.7.12 [46], with the settings “-x pacbio -B 3 -O 3,3 -L 7,7”. Alignment files were processed with SAMtools v1.9 [47]. For two isolates, *Leishmania donovani* FDAARGOS_361 and *Leptomonas pyrrhocoris* H10, the Illumina paired-end sequencing data were used to verify PacBio assemblies. Illumina reads were mapped with Bowtie2 v2.3.4.1 [48] with the following settings “-X 700 –very-sensitive –end-to-end –no-unal”. Coverage profiles were analyzed with SAMtools and custom python script, sorted bam files were inspected in IGB [49].

### 4.4. Analysis of Repeats

Frequency of each k-mer in a sequence was calculated using a custom python script. Repeated sequence regions were determined with the “repeat-match” from the MUMmer package. Regions with high level of sequence identity were found by the nucmer tool of the same package. Tandem repeats were identified using mreps v2.6 [50] with “-res 3 -minsize 40 -maxsize 6000 -minperiod 10 -maxperiod 4000” options. Inverted repeats were found with einverted from the EMBOSS package v6.6.0.0 [51] with “-match 3 -mismatch -4 -gap 12 -threshold 33”.

### 4.5. Plotting DR Maps

Resulting outputs of repeat and homology search algorithms were plotted using Circos [52]. Format conversion was performed with the python/awk scripts. The outer track of each Circos plot is a histogram showing the number of times a 24-mer, staring at the current position, appears in a maxicircle (24-mer frequency plot). Track min and max values were adjusted to show all 24-mers that appear 4–40 times (drawn with green colors), 24-mers with frequency value above 40 were also shown, but the height of the bars was fixed (drawn with blue). Tandem repeats were drawn as purple segments on the middle track of each Circos plot. The GC profiles (in window of 100 bp with a step of 1), were plotted in green on the inner track. Regions with sufficient sequence identity were drawn as colored ribbons, denoting the percent of sequence identity. Only regions over 100 bp and with minimal sequence identity of 80% were shown. The color of each ribbon was calculated by mapping sequence identity value in range [80, 100] to 11-class ‘Spectral’ color palette of ColorBrewer2 (http://colorbrewer2.org/#type=diverging&scheme=Spectral&n=9), which sets red, green, and blue colors for values around 80%, 90%, and 100%, respectively. Dotplot diagrams of maxicircles were generated with mummerplot in MUMmer v3.23.

## 5. Conclusions

We assembled and analyzed 17 complete maxicircles from different trypanosomatid species. Our analysis was focused on most arcane part of a maxicircle—the divergent region. This region is composed of repeated sequences, difficult to sequence and assemble, even with the modern NGS methods. Comparative analysis allowed us to infer the high-level architecture of the DR and describe some species-specific structural features. Our findings are consistent with previous works, which were focused on low-level sequence features, and shed light on how these low-level elements are organized and how they evolve.

## Figures and Tables

**Table 1:** Overview of assembled maxicircles. Brief statistics shows the basic assembly parameters: total maxicircle length, divergent region length, coding region length, average coverage per nucleotide, and total number of reads, included in the assembly and GenBank accession number.

Species (Strain)	Length, bp	DR, bp	CR, bp	Coverage	Reads	GenBank Accession
*Leishmania* (*Viannia*) *guyanensis* (204-365)	27,631	11,485	16,146	8.2	39	MN904521
*Leishmania (Leishmania) mexicana* (215-49)	27,138	10,860	16,278	10.6	36	MN904523
*Leishmania* (L.) *aethiopica* (209-622)	29,037	12,978	16,059	5.1	20 *	MN904514
*Leishmania* (L.) *infantum* (193-S1775)	29,512	13,313	16,199	21.7	94	MN904522
*Leishmania* (L.) *tropica* (216-162)	29,557	13406	16,151	20.1	64	MN904525
*Leishmania* (L.) *amazonensis* (210-660)	33,779	17,521	16,258	10.0	37	MN904515
*Leishmania* (L.) *donovani* (193-S616)	34,088	17,883	16,205	8.9	31	MN904519
*Leishmania* (L.) *donovani* (FDAARGOS_361)	33,278	17,238	16,040	16.0	61	MN904520
*Leishmania* (L.) *donovani* (Pasteur)	36,676	20,521	16,155	17.2	62	MN904518
*Leishmania* (V.) *braziliensis* (208-905)	26,728	10,568	16,160	13.9	89	MN904516
*Leishmania* (V.) *braziliensis* (208-954)	29,618	13,442	16,176	20.1	87	MN904517
*Trypanosoma brucei* (Lister 427)	23,201	8391	14,810	9.1	33 *	MN904526
*Trypanosoma cruzi* (TCC)	39,883	24,498	15,385	25.7	93	MN904528
*Trypanosoma cruzi* (Dm28c)	47,384	32,012	15,372	27.9	98	MN904527
*Leptomonas pyrrhocoris* (H10)	31,564	15,450	16,114	19.9	47	MN904524
*Herpetomonas megaseliae*	29,680	14,463	15,217	17.7	57	MN904513
*Crithidia expoeki* (BJ08_175)	25,722	8594	17,128	26.78	57	MN904512

* Assembled maxicircle contig was not marked as ‘circular’ by Canu assembler.

**Table 2:** Sources of used datasets. Accession numbers for raw data used to assemble maxicircle sequences in this study. All accessions numbers are for the PacBio reads, if not specified otherwise.

Species (Strain)	BioProject	Run Accession Number
*Leishmania* (L.) *mexicana* (215-49)	PRJNA484340	SRR7867272, SRR7867273, SRR7867284,SRR7867285
*Leishmania guyanensis* (204-365)		SRR7867261, SRR7867262, SRR7867269-SRR7867271
*Leishmania* (L.) *aethiopica* (209-622)		SRR8377733, SRR7867274, SRR7867278-SRR7867281
*Leishmania* (L.) *infantum* (193-S1775)		SRR7867264-SRR7867268
*Leishmania* (L.) *tropica* (216-162)		SRR7867286-SRR7867292
*Leishmania* (L.) *amazonensis* (210-660)		SRR7867275-SRR7867277, SRR8377732
*Leishmania* (V.) *braziliensis* (208-905)		SRR7880312; SRR7880319; SRR7880320
*Leishmania* (V.) *braziliensis* (208-954)		SRR7880309-SRR7880311
*Leishmania* (L.) *donovani* (193-S616)		SRR7880313, SRR7880314, SRR7880316
*Leishmania donovani* (1S line, FDAARGOS_361)	PRJNA231221	SRR5932752-SRR5932754, SRR5932751 (Illumina, paired-end)
*Leishmania donovani* (1S2D line, Pasteur) ^2^	PRJNA396645	SRR5902665-SRR5902672
*Trypanosoma brucei* (Lister 427)	PRJEB18945	ERR1794935-ERR1794915
*Trypanosoma cruzi* (TCC)	PRJNA432753	SRR6822075
*Trypanosoma cruzi* (Dm28c)	PRJNA433042	SRR6809376
*Leptomonas pyrrhocoris* (H10)	PRJNA598933 ^1^	
*Herpetomonas megaseliae*	PRJEB7883	ERR1036240-ERR1036242, ERR1046607-ERR1046611
*Crithidia expoeki* (BJ08_175)	Assembled sequence ^3^	

^1^ Sequence was assembled from PacBio data generated in our lab. ^2^ GenBank CP022652 (Ramasamy, G.; McDonald, J.; Sur, A.; and Myler, P., BioProject PRJNA396645, unpublished). ^3^ Sequence was assembled from PacBio data generated in [43].

## Supplementary Materials

The following are available online at https://www.mdpi.com/2076-0817/9/2/100/s1, **Figure S1**: Assembly quality control by read mapping on assembled contig. Screenshots from IGB with opened coverage tracks for bam files are shown. Red segment shows the borders of divergent region. (**a**) Mapping Illumina HiSeq paired-end reads (SRA: SRR5932751) with average insert size of 180 bp on *Leishmania donovani* strain FDAARGOS_361 maxicircle. (**b**) Mapping Illumina HiSeq paired-end reads with average insert size of 940 bp on *Leptomonas pyrrhocoris* H10 maxicircle. **Figure S2**: Comparison of *L. donovani* strain Pasteur assemblies. Assembly produced by our pipeline (with Canu) is on the left, maxicircle assembled with HGAP3 (GeneBank: CP022652) is on the right. Both assembled sequences are rotated to begin with the *ND5* gene (0 coordinate), so the upper part of the diagram corresponds to the CR and the lower one is the DR. Ribbon colors reflects the percent of sequence identity between regions in a range between 98% and 100%. Dark green corresponds to nearly 100% sequence identity, light green is the middle of interval (99%), red—identity near 98%. Only regions longer than 500 bp are shown.

## Appendix

Dotplots and Circos plots for species not included into the main text.
