# Supplementary material for: Common Structural Patterns in the Maxicircle Divergent Region of Trypanosomatidae
Source: Pathogens. 2020 Feb 5;9(2):100. doi: 10.3390/pathogens9020100 (PMC7169413; doi:10.3390/pathogens9020100)
Supplement: Supplementary file 1 [file pathogens-09-00100-s001.pdf]

Supplementary Materials

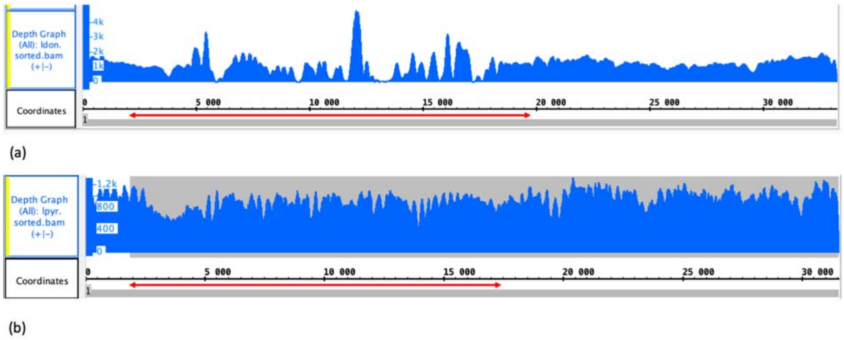

Supplementary Figure S1. Assembly quality control by read mapping on assembled contig. Screenshots from IGB with opened coverage tracks for bam files are shown. Red segment shows the borders of divergent region. (**a**) Mapping Illumina HiSeq paired-end reads (SRA: SRR5932751) with average insert size of 180 bp on *Leishmania donovani* strain FDAARGOS_361 maxicircle. (**b**) Mapping Illumina HiSeq paired-end reads with average insert size of 940 bp on *Leptomonas pyrrhocoris* H10 maxicircle.

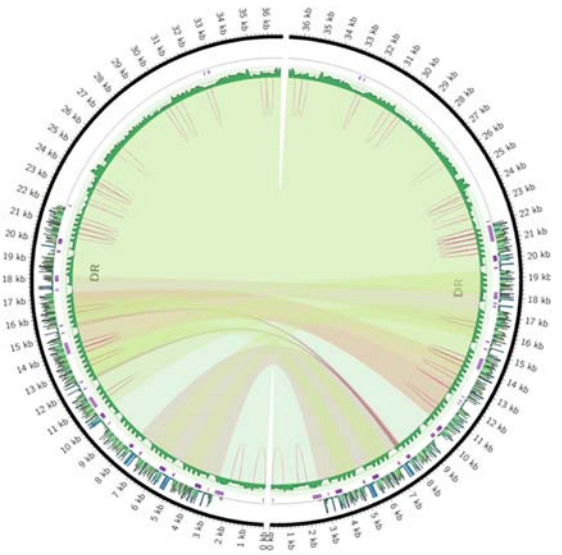

Supplementary Figure S2. Comparison of *L. donovani* strain pasteur assemblies. Assembly produced by our pipeline (with Canu) is on the left, maxicircle assembled with HGAP3 (GeneBank: CP022652) is on the right. Both assembled sequences are rotated to begin with the *ND5* gene (0 coordinate), so the upper part of the diagram corresponds to the CR and the lower one is the DR. Ribbons color reflects the percent of sequence identity between regions in a range between 98% and 100%. Dark green corresponds to nearly 100% sequence identity, light green is the middle of interval (99%), red – identity near 98%. Only regions longer than 500 bp are shown.
